# An analysis of gene/protein associations at PubMed scale

**DOI:** 10.1186/2041-1480-2-S5-S5

**Published:** 2011-10-06

**Authors:** Sampo Pyysalo, Tomoko Ohta, Jun’ichi Tsujii

**Affiliations:** 1 Department of Computer Science, University of Tokyo, Tokyo, Japan; 2 School of Computer Science, University of Manchester, Manchester, UK; 3 National Centre for Text Mining, University of Manchester, Manchester, UK

## Abstract

**Background:**

Event extraction following the GENIA Event corpus and BioNLP shared task models has been a considerable focus of recent work in biomedical information extraction. This work includes efforts applying event extraction methods to the entire PubMed literature database, far beyond the narrow subdomains of biomedicine for which annotated resources for extraction method development are available.

**Results:**

In the present study, our aim is to estimate the coverage of all statements of gene/protein associations in PubMed that existing resources for event extraction can provide. We base our analysis on a recently released corpus automatically annotated for gene/protein entities and syntactic analyses covering the entire PubMed, and use named entity co-occurrence, shortest dependency paths and an unlexicalized classifier to identify likely statements of gene/protein associations. A set of high-frequency/high-likelihood association statements are then manually analyzed with reference to the GENIA ontology.

**Conclusions:**

We present a first estimate of the overall coverage of gene/protein associations provided by existing resources for event extraction. Our results suggest that for event-type associations this coverage may be over 90%. We also identify several biologically significant associations of genes and proteins that are not addressed by these resources, suggesting directions for further extension of extraction coverage.

## Background

In recent years, there has been a significant shift in focus in biomedical information extraction from simple pairwise relations representing associations such as protein-protein interactions (PPI) toward representations that capture typed, structured associations of arbitrary numbers of entities in specific roles, frequently termed *event extraction*[1]. Much of this work draws on the GENIA Event corpus [2], a resource of 1500 PubMed abstracts in the domain of *transcription factors in human blood cells* annotated for genes, proteins and related entities, events and syntax [3-5]. This resource served also as the source for the annotations in the first collaborative evaluation of biomedical event extraction methods, the 2009 BioNLP shared task on event extraction (BioNLP ST) [6] as well as for the GENIA subtask of the second task in the series [7,8].

Another recent trend in the domain is a move toward the application of extraction methods to the full scale of the existing literature, with results for various targets covering the entire PubMed literature database of nearly 20 million citations being made available [9-12]. As event extraction methods initially developed to target the set of events defined in the GENIA / BioNLP ST corpora are now being applied at PubMed scale, it makes sense to ask how much of the full spectrum of gene/protein associations found there they can maximally cover. This issue is independent of the evaluation of the extraction performance of systems *for the associations they target*, addressed in the BioNLP ST and numerous other studies. Here, we will for simplicity assume that systems can eventually achieve satisfactory performance for associations for which annotated data is available. By contrast, we will assume that associations not appearing in this data cannot be extracted: as the overwhelming majority of current event extraction methods are based on supervised machine learning or hand-crafted rules written with reference to the annotated data, it reasonable to assume as a first approximation that their coverage of associations not appearing in that data is zero. In this study, we seek to characterize the full range of associations of specific genes/proteins described in the literature and estimate what coverage of these associations event extraction systems relying on currently available resources can maximally achieve. To address these questions, it is necessary not only to have an inventory of concepts that (largely) covers the ways in which genes/proteins can be associated, but also to be able to estimate the relative frequency with which these concepts are used to express gene/protein associations in the literature. Possible approaches to developing such an estimate include broad categories that could be characterized as “bottom-up” and “top-down”: either progressing from the unstructured natural language text toward the set of target concepts and their frequencies in the targeted expressions, or from a predefined set of concepts toward an estimate of these frequencies. As concepts relating to gene/protein associations are within the scope of many domain ontologies, most notably the community standard Gene Ontology (GO) [13], a top-down approach building on the identification of GO concepts in text is intuitively appealing. However, GO is intended for the annotation of gene/protein function and the structure of its terms removed from the way in which concepts are expressed in natural language text [14] and the recognition of concepts from ontologies such as GO in text is a challenging task where the reliability of available methods is limited [15]. Recognition performance is further likely to vary by concept depending on the ambiguity and variability of typical forms of expression (contrast e.g. *protein phosphorylation* with *protein binding*), leading to bias in frequency estimates. Finally, even given perfect recognition of concepts potentially expressing gene/protein associations it would remain necessary to determine which specific instances actually state such associations. We argue that when this determination is made, expressions stating the associations can be straightforwardly identified, making separate prior concept detection unnecessary. As a “bottom-up” approach is also more general in not relying on manually constructed resources, we chose to pursue such an approach in this work.

## Task definition

We term our extraction target *gene/protein associations.* So as not to limit the applicability of our results, we define our target entities (“genes/proteins”) broadly. The specific definition of this entity type applied in this study is provided by the GENETAG corpus annotation [16], as we make use of an automatic tagger trained on this resource for the recognition of genes/proteins. GENETAG annotates a single class of entities that encompasses genes and gene products (proteins and RNA) as well as related entities such as domains, promoters, and complexes. This inclusiveness permits the identification of associations between more than only the strict gene and gene product entities included in e.g. BioNLP ST annotation [4]. The corpus annotation includes a specificity constraint that excludes generic, non-named entity references such as *DNA sequence* from annotation, which is appropriate for our goal to identify associations of specific genes and proteins.

We also intend “associations” broadly, understanding it to encompass direct PPI-type interactions as well as experimental findings suggesting them (as targeted e.g. in the BioCreative PPI tasks [17]), BioNLP ST-style biomolecular events (“things that happen” involving genes/proteins) such as *expression* and *localization*, as well as *static relations*[*18*], associations such as *part-of* that hold between entities without necessarily implying change. Indeed, while we take “association” to exclude properties and states that involve only a single entity, we do not set other specific constraints, following instead a loose biologically motivated definition that can be characterized informally as “any association between genes, gene products, or related entities that is of biological interest.”

We note that while our aims and approach share a number of features with tasks such as protein-protein interaction extraction, they differ in focus on statements of association (as opposed to the entities stated to be associated) and in that we do not aim to reliably detect *instances* of the expressions of interest, but rather to estimate the distribution of association *types.* Due to the large scale of the PubMed corpus it is possible to pursue an approach that only considers a small, high-reliability portion of the available data (discarding most instances) and still identifies associations of interest. Thus, instead of instance-level extraction performance, we pay particular attention to not introducing overt bias e.g. toward particular forms of expression so as to be able to estimate relative frequencies of the associations in the full corpus.

## Corpus resources

**Figure 1:**
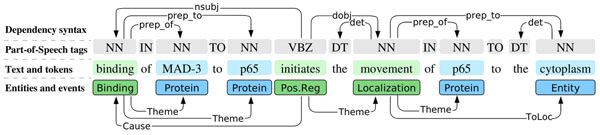
**Illustration of TPS corpus annotations for an example sentence.** Sentence splitting and constituency syntax not shown.

This study is based on the 2009 distribution of the full PubMed literature database, encompassing approximately 18 million citations of biomedical domain scientific articles. For the analysis of this data, we make use of the Turku PubMed Scale (TPS) corpus [10], a corpus covering the entire PubMed automatically annotated for sentence boundaries, gene/protein named entities, sentence syntax (both constituency and dependency), and events. Figure 1 illustrates these annotations. Note that while the original focus of the corpus is on BioNLP ST events, we ignore the event annotations of the corpus. Instead, we make use of the automatic annotations originally created for supporting the extraction of the events, briefly presented in the following.

All PubMed documents in the TPS corpus were initially processed with the GENIA sentence splitter with simple heuristic post-processing to correct some errors from the machine learning-based splitter [19]. The sentence splitter is estimated to achieve an F-score of 99.7% on the GENIA corpus. Gene/protein named entities were tagged in all sentences using the BANNER named entity recognition system [20] trained on the GENETAG corpus [16] and thus reflect its inclusive definition of gene/protein (as discussed above). The release of BANNER applied to tag the TPS corpus was reported to achieve 86.4% F-score on the GENETAG corpus, and an evaluation on a random sample of tagged entities in TPS data found 87% precision [21], suggesting that the tagger generalizes well to the whole PubMed.

Finally, the TPS corpus distribution includes syntactic analyses for all sentences in which at least one named entity has been tagged. (Sentences not containing entities are not parsed as parsing was the most computationally intensive part of the automatic corpus annotation and the event extraction system could only extract events from sentences containing entities.) Parses were produced using the McClosky-Charniak parser [22], a version of the Charniak-Johnson parser [23] adapted to the biomedical domain. The parser has shown state-of-the-art performance in recent intrinsic [22,24] and extrinsic [25,26] evaluations. The McClosky-Charniak parser produces constituency (phrase structure) analyses in the Penn Treebank scheme, with Penn part-of-speech tags. In addition to the these analyses, dependency analyses in the Stanford Dependency (SD) scheme [27], created from the constituency analyses by automatic conversion using the using the Stanford parser tools [28] (Version 1.6.1) are provided in the TPS corpus. In addition to the TPS corpus, we use the BioNLP ST 2009 data [6] for training the statistical component of our method and for one aspect of the evaluation, as described in detail in the sections on Machine Learning and Evaluation.

## Identification of gene/protein associations

In this section, we present our approach to identifying statements of gene/protein associations. We assume throughout that gene/protein associations are stated through specific words, analogously to the widely applied concepts of *interaction words* in protein-protein interaction extraction and *trigger* (or *text binding*) words in event extraction. We follow a statistical approach to identifying such candidate words, introduced in the following through an extended analysis of word statistics in PubMed.

### Overall statistics

As expected for a corpus of English, the most frequent words in PubMed are prepositions, determiners, conjunctions, forms of the copula (“is”, “are” etc.) and, if non-word tokens are included, punctuation. In this work, we focus on content words, filtering closed class words and non-words and applying a basic stopword list including the PubMed stopwords [29]. Table 1 shows the most frequent such words in PubMed. For this and other word statistics in this section, basic tokenization separating punctuation from words and lowercasing has been applied but stemming or lemmatization is not performed. The distribution suggests that medical topics dominate biomolecular ones overall, with e.g. the word “patients” occurring more than three times as often as the word “protein”. Although general expressions such as the included “activity” and “effect” can be used to describe gene/protein associations, this list contains no word specific to such associations.

**Table 1:** Most frequent words in PubMed

Word	Frequency
patients	8728330
cells	5384960
results	4175016
study	4149760
treatment	3436331
cell	3230831
activity	2763031
group	2635275
protein	2553732
effect	2457417

### Gene/protein mentions

The automatic tagging for mentions of gene/protein entities in the TPS corpus covers a total of 36.4 million gene/protein mentions in 5.4 million documents, approximately 30% of all PubMed citations. These annotations allow focus on texts likely relevant to gene/protein associations. Here, as we are interested in particular in texts describing associations between two or more gene/protein related entities, we apply a focused selection, picking only those individual sentences in which two or more mentions co-occur. While this excludes associations in which the entities occur in different sentences, their relative frequency is expected to be low: for example, in the BioNLP ST data, all event participants occurred within a single sentence in 95% of the targeted biomolecular event statements. Based on our experience with event annotation, we further expect that in a corpus of this size the great majority of association types that are expressed across multiple sentences in some statements will also appear within a single sentence in others. In the TPS data, there are 9.0 million sentences with at least two tagged gene/protein entities. These sentences contain 25.4 million entity mentions; approximately 70% of the corpus total. Table 2 shows the most frequent words in sentences with at least two tagged protein mentions. The list suggests that this simple selection is sufficient to identify a subset of PubMed where biomolecular topics are prominent: both “protein” and “expression” appear ranked near the top.

**Table 2:** Most frequent words in sentences containing two or more gene/protein entity mentions in PubMed

Word	Frequency
cells	1455897
protein	1057920
expression	923002
activity	753521
cell	750293
gene	704434
receptor	641766
human	635468
levels	603117
factor	518676

### Dependency paths

The TPS corpus contains both constituency and dependency analyses of sentence syntax for all sentences with at least one gene/protein mention. While both forms of representation arguably capture largely the same information, dependency representations have been argued to make the relevant syntactic relations more immediately accessible and have been successfully employed in many recent domain information extraction approaches, frequently in conjunction with the use of the *shortest dependency path* between two entities to discover stated associations (see e.g. [30-33]).

**Figure 2:**
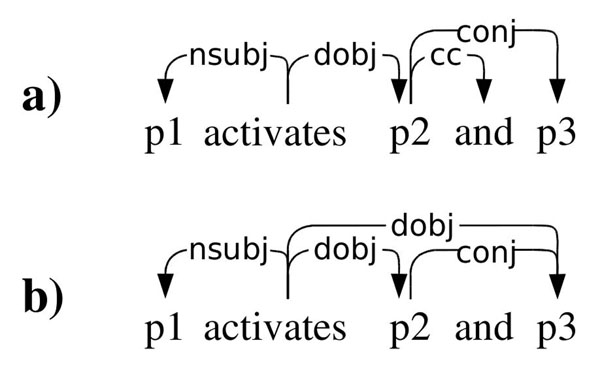
**Variants of the Stanford Dependency representation**. a) Basic representation. b) Collapsed, coordination-processed representation.

Here, we follow the assumption that when two entities are stated to be associated in some way, the most important words expressing their association will typically be found on the shortest dependency path connecting the two entities (cf. the *shortest path hypothesis* of Bunescu and Mooney [30]). The specific dependency representation applied here is the collapsed, coordination-processed variant of the Stanford representation, which is expressly oriented toward use in this type of information extraction approaches [27]. When extracting the shortest paths, we further avoid traversing coordinating conjunction dependencies (conj*) to assure that relevant words are not excluded in sentences involving coordination and that similar paths are extracted for all coordinated words (Figure 2).

The corpus contains 31.8 million pairs of gene/protein mentions co-occurring in a sentence, and a connecting shortest path could be extracted for 97% of these (failures to extract a path were primarily due to clause-level coordination – e.g. “we study P_1_ and we find that P_1_ is ...” – and, rarely, failures from the parser or the dependency conversion). Table 3 shows the words most frequently occurring on these paths. This list again suggests an increased focus on words relating to gene/protein associations: *expression* is the most frequent word on the paths, and *binding* appears in the top-ranked words.

**Table 3:** Most frequent words on shortest dependency paths connecting two gene/protein entity mentions in PubMed

Word	Frequency
expression	590810
activity	470393
levels	386130
cells	349648
activation	240942
induced	221177
binding	153806
mediated	129620
effect	124948
increased	124564

### Path probabilities

Entities often co-occur in text without any association being stated between them, but some shortest dependency path can be found connecting (nearly) all co-occurring entities. Distinguishing paths that state associations from those that do not could thus help identify words that are key to expressing those associations.

A wealth of approaches for distinguishing relevant paths from irrelevant ones have been proposed in the protein-protein interaction extraction literature, including rule-based, pattern-based (hand-written and learned) and supervised classification-based methods (e.g. [31,32,34-38]). However, writing explicit rules conflicts with our aim of discovering associations (and statements of associations) that we do not already know about, and application of standard supervised learning methods would similarly limit the scope of what can be extracted by the (known) training data.

Here, drawing in part on ideas from Open Information Extraction [39], we adopt a probabilistic approach using an “unlexicalized” machine learning method. We defer detailed description of the method to a later section (Machine Learning), now simply assuming a way to assign to each path *p* an (estimated) probability *P*(*p*) that the path expresses an association between the entities it connects. We make use of *P*(*p*) in two obvious ways to refine the pure frequency-based word rankings presented above: first, only count words when they occur on paths that have an estimated probability higher than a given threshold of being relevant, and second, replacing the “raw” word count with the expected number of times that word appears in a relevant path, informally *E_w_* = *∑_p:w∈p_P*(*p*).

Table 4 shows the top-ranked words by *E_w_* as calculated using the method described below. We find in this listing only words that are regularly used to express gene/protein associations, suggesting that probabilistic ranking can allow clear focus on the targeted statements.

**Table 4:** Words ranked highest by *E_w_*, the expected number of times they occur on shortest paths likely to express a gene/protein association

Word	*E_w_*
expression	68803.3
activity	56372.9
activation	43987.9
binding	28989.3
induced	24132.8
phosphorylation	22971.9
binds	17757.0
production	16893.2
inhibited	15972.9
inhibition	14546.0

## Machine learning

We applied supervised machine learning to estimate the probability that a dependency path connecting two gene/protein named entity mentions expresses an association of these entities, training with “unlexicalized” features [40] to force the learning method to generalize and to learn based on the patterns of expression only.

### Training data

For training data, we could potentially draw from a wealth of corpus resources annotated for some form of association between genes/proteins, such as PPI corpora (see e.g. [41]). However, as we are in particular interested in event extraction approaches, we chose to use the BioNLP ST 2009 data (the BioNLP ST 2011 datasets were not available when this work was performed). This dataset also identifies the expressions stating the annotated events (“trigger words”), providing test material for the method.

**Figure 3:**
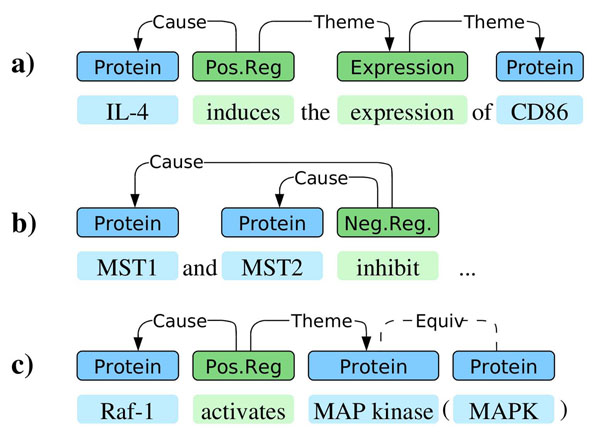
**Reinterpreting BioNLP Shared Task event structures as associated entity pairs**. A positive pair is extracted for the proteins in a) but not in b) as there is no causal connection leading from one to the other. In c), two positive pairs, (*Raf-1*,*MAP kinase*) and (*Raf-1*,*MAPK*), are extracted due to the equivalence relation.

As the BioNLP ST data does not explicitly identify simple *pairs* of entities that are stated to be associated (but rather event graphs), it was first necessary to derive a pairwise representation from the event representation. We applied a mapping similar to that introduced by [42] for deriving pairwise relations from the event-style annotations of the Biolnfer corpus [43]: for each co-occurring entity pair, we identified all paths through event structures connecting the two entities. If these paths included at least one where the direction of causality was not reversed on the path, the pair was marked as a positive example of an association; otherwise it was marked negative. Finally, we interpreted the Equiv annotations identifying equivalent entity references in the data: any pair where entities are equivalent to those of at least one positive pair was marked positive (see Figure 3).

Finally, to make this pair data consistent with the TPS event spans, tokenization and other features, we aligned the entity annotations of the two corpora. Alignment was necessary in particular for entities as the GENETAG corpus annotation criteria differ notably from those of the BioNLP ST data, which only annotates specific gene and gene product names and not, for example, protein domains or complexes [44]. We mapped a BioNLP ST entity to a TPS entity if their spans matched or the source entity was entirely contained within the span of the candidate target entity. Unmatched entities were removed from the data. This processing was applied to the BioNLP ST training set, creating a corpus of 6889 entity pairs of which 1119 (16%) were marked as expressing an association (positive).

### Learning method

**Figure 4:**
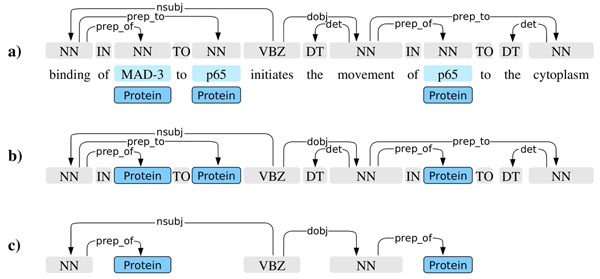
**Unlexicalized shortest path representation** a) Applied annotations with original sentence text. b) Unlexicalized representation. c) Shortest path connecting two gene/protein mentions.

We applied the libSVM Support Vector Machine implementation using probabilistic outputs [45]. For training the classifier, we applied features derived only from the words and dependencies along the shortest path between any two entities. We first replaced each word marked as a gene/protein mention with a placeholder string and each other word with its part of speech tag, using the Penn tags included in TPS data (Figure 4). We then generated a set of frequently used dependency path features from this representation (see e.g. [32,33,38,46]): path length, path “tokens” (PoS/placeholder), dependency types on the path, and “token”/dependency 2-grams and 3-grams. Preliminary experiments using cross-validation on the training data suggested performance was not sensitive to the details of the feature representation. The SVM regularization parameter was selected similarly, testing parameter values on the scale ..., 2^–1^, 2^0^, 2^1^,... and selecting *c* = 2^–3^ for the final experiment.

The resulting classifier is intentionally weak, being trained to recognize not the specific properties of positive examples in its training set but rather their general characteristics. Development testing indicated an F-score and AUC of approximately 50% and 70%, substantially below the state of the art for the comparable PPI pair extraction task [32] as expected.

### Calculating *E_w_*

*E_w_*, informally characterized as the expected number of times a word *w* occurs on a dependency path which is estimated to be likely to express a gene/protein association, is central to the applied probabilistic ranking. In technical detail, we derived *E_w_* as follows.

We first extracted all instances of shortest dependency paths connecting two genes/proteins. We then combined all paths sharing the same “unlexicalized” representation, giving a total of 6.8 million unique paths. To make storage and processing more feasible, we removed paths occurring only once in the entire corpus. This filtered out 6.0 million paths – 88% of the total number of unique paths – but due to the Zipfian properties of the distribution, the remaining 0.8 million unique paths account for 16.7 million occurrences, or 74% of the total occurrences. We thus do not expect this practically motivated filtering to fundamentally alter the basic statistical properties of the data.

Each path was then assigned the estimated probability *P*(*p*) using the probabilistic outputs of the SVM trained as described above. At this stage, we could potentially introduce a threshold parameter into the method defining a tradeoff between path quality and inclusiveness. However, as initial testing suggested the method to be relatively robust to the choice of cutoff, we simply take the obvious choice of defining as “likely positive” path any for which *P*(*p*) > 0.5. We then removed any path that did not meet this condition as not likely expressing an association, leaving 46437 unique unlexicalized paths (5.7% of the total) predicted to express gene/protein associations. Finally each occurrence of a word *w* on one of these paths is assigned the path probability *P*(*p*)*.* In cases where words appear on multiple paths, they are simply assigned the maximum of the path probabilities. *E_w_* is then the sum of these probabilities over the entire corpus.

We note that this formulation does not include any normalization by the overall frequency of words. This implies that high-frequency irrelevant words (such as “gene”) are likely to receive higher *E_w_* values than rare relevant words (such as “biotinylation”). However, normalization was not included as it would reduce the ability to use the results to estimate the relative frequency of the words in relevant expressions. For efforts aiming only to discover new expressions of entity associations without regard to their frequency, we expect incorporation of some form of correction by the overall frequency of words would be beneficial.

## Evaluation

**Figure 5:**
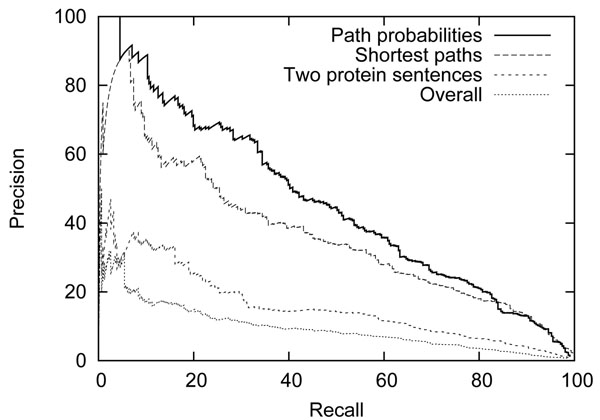
Precision/recall curves of the four word rankings for BioNLP ST trigger words

We first evaluated each of the word rankings discussed in the section on Identification of Gene/Protein Associations by comparing the ranked lists of words against the set of single words marked as trigger expressions in the BioNLP ST development data. These single-word triggers account for 92% of all trigger expressions marked in the data, and there are 343 unique triggers. Figure 5 shows precision/recall curves for each of the four rankings generated by the word frequency/expected value. The result supports the informal observations made through the top-ranked words in Tables 1, 2, 3 and 4: the later approaches provide a much more relevant ranking for identifying words expressing associations.

To evaluate the capability of the presented approach to identify new expressions of gene/protein associations, we next performed a manual study of candidate words for stating gene/protein associations using the *E_w_* ranking. Here, we take as *known* any word for which the normalized, lemmatized form (using the NLM LVG norm normalizer [47]) matches that of any word appearing as a trigger expression in the BioNLP ST training or development test data. We then selected the words ranked highest by *E_w_* that were not known, grouped by normalized and lemmatized form, and added for reference examples of frequent shortest dependency paths on which any of these words appear (see example in Table 5). These groups were evaluated by a PhD biologist with expertise in event annotation and basic understanding of the Stanford Dependency representation of syntax (TO), with instructions to mark as positive words that in contexts like those provided can be understood to express a gene/protein association, defined broadly as described in the Task Definition section.

**Table 5:** Example shortest paths for candidate gene/protein association-expressing word “acylation”

GGP	<prep_of acylation prep_by>	GGP
GGP	<hyphen dependent <amod acylation prep_of>	GGP
GGP	<nsubj stimulated dobj> acylation prep_of>	GGP
GGP	<prep_of acylation prep_by> GGP appos>	GGP
GGP	<nsubj decreased dobj> acylation prep_of>	GGP

In total, 1200 candidate expressions were manually evaluated, proceeding from candidates ranked highest by *E_w_* to lower. While no stopping criterion was specified in advance, evaluation was stopped after reaching a point of diminishing returns where no positives had been identified in a run of over 100 examined candidates. This process necessarily misses relevant types of associations in the “long tail” of the distribution, but they are expected to be rare: for illustration, the lowest-ranked positive event-type association word “biotinylation” has an *E_w_* value of 42.3; by contrast, “phosphorylation” (the most frequent post-translational modification) has an *E_w_* of 35708.2, suggesting the latter is several orders of magnitude more common as an expression of gene/protein association. (Note that these values differ from those in Table 4 as they include variants that are lemmatized to the same string.)

**Figure 6:**
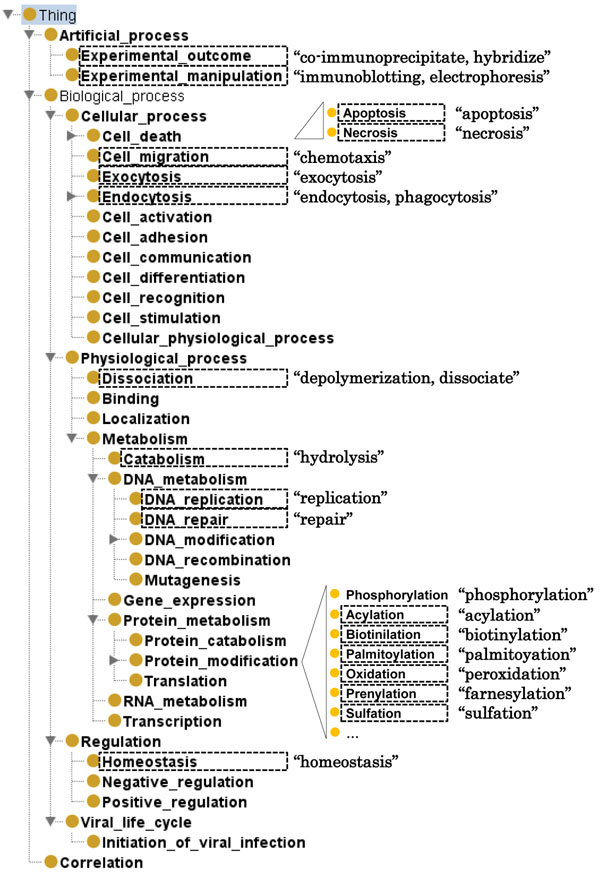
**Organization of proposed new event classes into the GENIA ontology.** New classes shown as dotted rectangles with examples of expressions stating each type.

Of the examined candidates, 660 were judged as positive in total, confirming that the approach can identify expressions of entity associations not appearing in the reference annotated data. We next proceeded to manually cluster these by the type of association they would typically be expected to express. Following preliminary analysis, we performed a top-level division into three categories: events (“things that happen”) involving gene/protein entities in their natural environment (55% of associations), “static” relations holding between the entities (28%), and experimental observations and manipulations that do not occur naturally (17%). (Note that these numbers are on the level of association types and do not take into account the number of instances of each type.) We further grouped the new event statements into event classes using the Gene Ontology [13] for reference and identified event classes that were not previously included in the GENIA event ontology. This process suggested 18 event classes that were not previously considered in GENIA resources, shown in Figure 6 with a tentative proposal on how these classes could be organized into the GENIA ontology and examples of identified words expressing each new event type. It should be noted that while these classes are new to the GENIA ontology, they could be found in other ontologies, again notably GO. However, as GO contains more than 20,000 biological process terms, purely manual identification of terms specifically relevant to frequent associations of entities of interest would require considerable effort.

**Figure 7:**
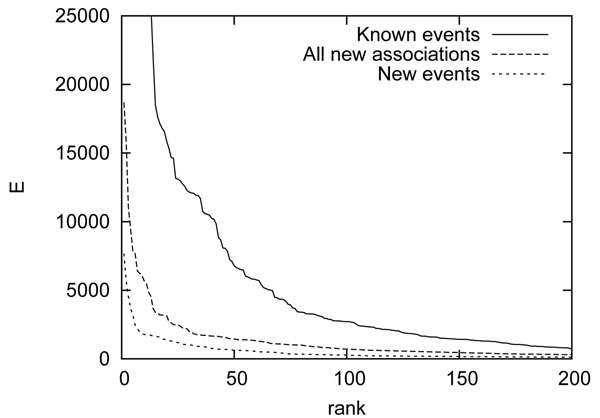
**Comparison of estimated coverage of previously known and newly identified words expressing gene/protein associations.** Note truncated ranges.

Finally, to estimate the relative prominence of the known (i.e. BioNLP ST) expressions of associations in PubMed compared to those that were newly identified, we compared the *E* values of the unique lemmas, counted as the sum of *E_w_* for words sharing the lemma. Figure 7 shows a plot of the values ranked from high to low *E.* The result was unexpected: the estimate suggests that even though the newly identified association words are drawn from PubMed without subdomain restrictions and include more than only event expressions, expressions of event-type associations using the previously known words are overall much more prominent in PubMed. Specifically the total *E* value mass of all the newly identified associations (the area under the curve in Figure 7) is just 22% of that of the known events, and the mass of the newly identified events 37% of all the new associations; only 8% of that of the known events. If static relations and experimental observations and manipulations are excluded as (arguably) not in scope for event extraction, this estimate suggests that currently available resources for event extraction cover over 90% of all events involving gene/protein entities in PubMed.

## Discussion

We found that out of all gene/protein associations in PubMed, currently existing resources for event extraction are lacking in coverage of a number of event types such as *dissociation*, many relatively rare (though biologically important) protein post-translational modifications, as well as some high-level process types involving genes/proteins such as *apoptosis.* In addition to event types, associations characterized as experimental outcomes and manipulations and static relations (e.g. *part-of*) were prominent among those not covered by the considered resources. Only the first of these categories is unambiguously within scope for event extraction. However, while statements of experimental results such as *colocalize* and *coprecipitate* do not directly state a biologically meaningful association between genes/proteins, they suggest a possible association and have been specifically included in a number of tasks targeting protein-protein interactions, including BioCreative challenges [17]. This suggests that for practical applications it may be important to consider also this class of associations. Likewise, while static relations are (by definition) not events and rarely primarily targeted in domain information extraction studies, the analysis suggests they are relatively frequent among gene/protein associations not covered by the considered resources, and they have been argued to play a potentially important supporting role in event extraction [18].

Despite these areas of missing coverage, the statistical analysis suggests that resources already cover the clear majority of gene/protein events in PubMed, indicating that annotation-based approaches to extending coverage of event types (e.g. [48-51]) may offer a realistic path to near-complete coverage of all major gene/protein events in the near future. With resources for static relation extraction this coverage can be further extended beyond event-type associations, for example applying static relations in support of event extraction as considered in the REL task of BioNLP Shared Task 2011 [52].

While these results are highly encouraging, it must be noted that the approach to identifying gene/protein associations considered here is limited in a number of ways: it excludes associations stated across sentence boundaries and ones for which the shortest path hypothesis does not hold, does not treat multi-word expressions as wholes, ignores ambiguity in implicitly assuming a single sense for each word, and only directly includes associations stated between exactly two entities. The approach is also fundamentally limited to associations expressed through specific words and thus blind to e.g. part-of relations implied by statements such as *CD14 Sp1-binding site.* Further, our estimate of overall association statement frequency ignored much of the “long tail” of the distribution, thus excluding rare expressions which may nevertheless add up to a not insignificant fraction of the total. These factors limit the reliability of the presented coverage estimates. Mitigation or elimination of these factors remains future work. Finally, it should be recalled that while we have taken any expression of association for which even a single annotated instance exists as “known”, the performance at which many of these association can be extracted in practice may be limited.

## Conclusions

We have presented an approach to discovering expressions of gene/protein associations from PubMed based on named entity co-occurrences, shortest dependency paths and an unlexicalized classifier to identify likely statements of gene/protein associations. Drawing on the automatically created full-PubMed annotations of the Turku PubMed-Scale (TPS) corpus and using the BioNLP’09 shared task data to define positive and negative examples of association statements, we distilled an initial set of over 30 million protein mentions into a set of 46,000 unique unlexicalized paths estimated likely to express gene/protein associations. These paths were then used to rank all words in PubMed by the expected number of times they are predicted to express such associations, and 1200 candidate association-expressing words not appearing in the BioNLP’09 shared task data evaluated manually. Study of these candidates suggested 18 new event classes for the GENIA ontology and indicated that the majority of statements of gene/protein associations not covered by currently available resources are not statements of biomolecular events but rather statements of static relations or experimental manipulation.

The event annotation of the GENIA corpus was originally designed to cover events discussed in publications on a limited subdomain of biomolecular science. It could thus be assumed that the event types and the specific statements annotated in GENIA would have only modest coverage of all gene/protein association types and statements in PubMed. However, our results suggest that even the BioNLP’09 shared task data, a subset of GENIA, may represent a clear majority of all gene/protein associations. This estimate of coverage is a first attempt and involves many uncertain factors and potential sources of error, calling for more research.

The data derived from TPS created in this study, including the shortest paths, their estimated probabilities, and the word lists ranked by probability of stating a gene/protein association are available for research purposes from from the GENIA project homepage http://www-tsujii.is.s.u-tokyo.ac.jp/GENIA.

## Competing interests

The authors declare that they have no competing interests.

## Authors’ contributions

SP and TO conceived of the study. SP designed and implemented the experiments and drafted the manuscript. TO performed the manual evaluation and analysis and helped draft the manuscript. JT participated in the study design and coordination and helped draft the manuscript. All authors read and approved the final manuscript.
